# Sad Casuality

**Published:** 1853-07

**Authors:** 


### SAD CASUALITY.

"On Thursday last, Mr. Edward Scanlin, Druggist, near the corporation line in Fulton, had three teeth upon a gold plate placed in his mouth. Tuesday night while asleep the plate became loose and Mr. S. swallowed it a short distance below the palate, when the suffocation it occasioned caused him to awake. He could not get it up nor down and thus stationary, it gave him much pain. The anterior portion of the neck became swollen and it was thought that lock-jaw would ensue. Dr. Mussey was sent for who yesterday succeeded in removing the teeth and Mr. Scanlin may now recover. Yesterday morning his case was considered hopeless."

The above is taken from one of our daily papers, and one of the physicians who saw the case informs us that there was no just cause for serious alarm. Some irritation of the throat was occasioned, which however soon subsided after the teeth were forced into the stomach. We do not know whether they are yet digested, nor do we know who furnished the gentleman with so indigestable a meal.

